# *Parasites & Vectors*: 13 years devoted to parasitology and tropical medicine

**DOI:** 10.1186/s13071-021-04965-2

**Published:** 2021-09-01

**Authors:** Filipe Dantas-Torres

**Affiliations:** Aggeu Magalhães Institute, Oswaldo Cruz Foundation (Fiocruz), Recife, Brazil

*Parasites & Vectors* (or just *P&V*) was officially launched in 2008 [1], also incorporating two previous BioMed Central journals, *Filaria Journal* and *Kinetoplastid Biology and Disease*. As an author, I liked *P&V* at first sight. The main features of the new journal were fast processing of manuscripts and fair refereeing and editorial decision-making. More than that, Prof. Chris Arme, the founding Editor, created a new community, nurturing the spirit of collaboration between authors, reviewers, handling editors and publisher. This was remarkable and positively affected the publishing experience of authors, from initial submission to the final online publication. I experienced this myself as an author back in 2008 [2], when I published my first paper in *P&V*.

During the past 13 years, *P&V* has emerged as a leading journal, publishing high-quality manuscripts on parasites, vectors and vector-borne diseases. Incidentally, in the new Journal Citation Reports, the impact factor of *P&V* increased from 2.824 (2019) to 3.876 (2020)! This is an outstanding achievement and places *P&V* among the top journals (quartile 1) in the field of parasitology and tropical medicine. This achievement is certainly a result of integrated efforts from authors, reviewers, editors and journal staff. This also highlights that *P&V* is a well-regarded journal by the scientific community and that manuscripts published in this journal are highly cited.

I have been involved with *P&V* activities since its foundation. In this sense, I am indebted to Prof. Chris Arme and Prof. Aneta Kostadinova, former Editors-in-Chief, for the numerous opportunities given to act as reviewer, associate editor and subject editor. In November 2020, I became the Editor-in-Chief of *P&V*, which is a great honour and a great responsibility.

During the past months, we refreshed the sections and the team of Subject and Associate Editors. The journal is currently structured with the following sections:

- Dipteran vectors and associated diseases (edited by Fredros Okumu, Ifakara Health Institute, Tanzania, and Marco Pombi, La Sapienza University of Rome, Italy);
- Helminths and helminthic diseases (edited by Martin Walker, Royal Veterinary College and Imperial College London, UK);
- Parasite genetics, genomics and proteomics (edited by Xing-Quan Zhu, Shanxi Agricultural University, China);
- Parasites of veterinary importance (edited by Adnan Hodžić, University of Veterinary Medicine, Austria);
- Protozoa and protozoan diseases (edited by Anna Bajer, University of Warsaw, Poland);
- Ticks and tick-borne diseases (edited by Martin Pfeffer, University of Leipzig, Germany).

The full list of Associate Editors and more information about each section is available at our homepage (https://parasitesandvectors.biomedcentral.com/manuscripts/sections). Submitting authors may feel that their manuscripts could fit in one or more sections, and this is okay. For instance, a manuscript reporting a study on *Leishmania* parasites might fit best in the section Protozoa and protozoan diseases (e.g., studies on *Leishmania* biology), Dipteran vectors and associated diseases (e.g., studies on *Leishmania* transmission and sand flies), Parasites of veterinary importance (e.g., studies on animal leishmaniasis) or even Parasite genetics, genomics and proteomics (e.g., studies on *Leishmania* genetics and genomics). So, submitting authors should choose the most appropriate section according to the main focus of their manuscripts.

We have also recently published a new Editorial Style Guide (available at https://parasitesandvectors.biomedcentral.com/submission-guidelines). The objective is to provide detailed information for the submitting authors to prepare their manuscripts according to the journal style and standards. We believe this will expedite the production time and increase the overall quality of the published papers. Springer Nature also offers a number of author services, including academic translation, manuscript formatting and figure services (for more information, see https://authorservices.springernature.com). I strongly recommend these services for authors needing English editing and also technical support to prepare high-quality figures, for instance. This will directly and positively impact on the quality of their published papers.

We have also slightly changed the scope of *P&V* (for more information, see https://parasitesandvectors.biomedcentral.com/about). We welcome high-quality manuscripts on all aspects of the basic and applied biology of parasites, parasitic diseases, intermediate hosts, vectors and vector-borne pathogens. Besides traditional and well-established areas of science in these fields, we aim to provide a platform for publication of the rapidly developing resources and technology in parasite, intermediate host and vector genetics, genomics, proteomics. Manuscripts addressing broader issues, such as economics, social sciences and global climate change in relation to parasites, vectors and parasitic disease control, are also considered. If you are unsure whether your paper is in scope for *P&V*, you can submit a pre-submission enquiry, providing an abstract of your work. I will be pleased to provide you a timely response.

As Editor-in-Chief, I shall ensure that *P&V* will continue to serve as a platform for scientists from all over the world to publish their manuscripts reporting new developments in the field of parasites and vectors. Last but not least, I will continue to nurture the spirit seeded 13 years ago by Prof. Chris Arme, to foster the collaboration between authors, reviewers, editors and journal staff.

## References

[1] Arme C (2008). Welcome to parasites & vectors. Parasit Vectors.

[2] Dantas-Torres F (2008). Canine vector-borne diseases in Brazil. Parasit Vectors.

